# IL-6–Caspase 3 Axis Plays an Important Role in Enteritis Caused by *Legionella pneumophila* Pulmonary Infection

**DOI:** 10.3390/microorganisms13020313

**Published:** 2025-02-01

**Authors:** Dahui Zhao, Xuefeng Duan, Li Zhu, Min Fang, Tian Qin, Yuhai Bi

**Affiliations:** 1 CAS Key Laboratory of Pathogen Microbiology and Immunology, Institute of Microbiology, Center for Influenza Research and Early-Warning (CASCIRE), CAS-TWAS Center of Excellence for Emerging Infectious Diseases (CEEID), Chinese Academy of Sciences, Beijing 100101, China; zhaodah@163.com (D.Z.); duanxf@im.ac.cn (X.D.); zhuli3210@gmail.com (L.Z.); 2 University of Chinese Academy of Sciences, Beijing 100049, China; 3 School of Life Sciences & Henan Key Laboratory of Synthetic Biology and Biomanufacturing, Henan University, Kaifeng 475004, China; fangmin@henu.edu.cn; 4 State Key Laboratory for Infectious Disease Prevention and Control, National Institute for Communicable Disease Control and Prevention, Chinese Centre for Disease Control and Prevention, Beijing 102206, China

**Keywords:** *Legionella pneumophila*, enteritis, inflammatory cytokines, caspase 3, IL-6

## Abstract

Background: Since *Legionella pneumophila* (Lp) is widely present in natural and artificial water environments, it has a high potential risk of outbreak. Diarrhea caused by Lp pulmonary infection is an important symptom of Legionnaires’ disease (LD); however, the underlying mechanism of the diarrhea has not yet been revealed. This not only has a negative impact on clinical diagnosis and treatment, but may also cause misdiagnosis. Methods: In the present study, a mouse model of enteritis caused by pulmonary infection of Lp was established. By using this mouse model, we explored the underlying mechanisms of the enteritis caused by Lp pulmonary infection. Results: The results indicated that the systemic inflammatory response played a very important role in the enteritis phenotype caused by a strong-virulence strain of Lp. Furthermore, we found that the expression of Bcl-2 was downregulated by IL-6 through the p53 signaling pathway, thereby activating the caspase 3 of intestinal epithelial cells (IECs), causing the apoptosis of IECs, and ultimately leading to the enteritis phenotype. Conclusions: The IL-6–caspase 3 axis plays an important role in enteritis caused by Lp pulmonary infection.

## 1. Introduction

*Legionella* is the pathogen that causes Legionnaires’ disease (LD). It was first discovered while studying the pathogen that caused the pneumonia that broke out at the 1976 Veterans Conference in Philadelphia, USA [1]. *Legionella* is very widely distributed in almost all natural water environments (such as streams, rivers, lakes, and hot springs), moist soils, and mires. In addition, because *Legionella* can survive chlorination, it can enter water supply systems (e.g., cooling towers for air conditioners, air conditioners, hot water systems, shower heads, faucets, and ventilation fans) and multiply when conditions are suitable [2]. To date, more than 70 species of *Legionella* have been identified [3]. All identified species have been successfully isolated from the environment, and about 30 of them infect humans, mainly in the lower respiratory tract. *Legionella* infections can cause pneumonia or a milder form of fever called Pontiac fever. More than 90% of all *Legionella* infections are caused by *L. pneumophila* (Lp) [4]. *Legionella* infection is mainly caused by the inhalation of aerosols containing the bacteria. Other less common routes include the inhalation of contaminated water and direct contact with a wound. Notably, there has also been a report of suspected direct human-to-human transmission [5]. Factors such as smoking, aging, chronic cardiovascular or respiratory diseases, diabetes, alcoholism, cancer, and immunodeficiency can lead to an increased likelihood and symptoms of *Legionella* infection. When Lp is coinfected with viruses, such as influenza virus, it will become more virulent, leading to more severe symptoms [6]. In addition, under some special conditions, the chance of a *Legionella* infection may increase. For example, during the SARS-CoV-2 pandemic, the indoor temperature of some buildings in the United States rose due to the prolonged lockdown, coupled with the lack of sufficient replenishment of disinfectants in the water, resulting in a large amplification of *Legionella* bacteria, which greatly increased the risk of *Legionella* outbreaks [7]. LD is grossly underestimated in many countries due to the lack of routine surveillance and detection systems [8]. Due to the widespread presence of *Legionella* in natural and artificial water environments, *Legionella* poses a potentially severe threat to humans.

Both innate and acquired immunity play important roles in the control of Lp infection. The activation of innate immune cells relies mostly on the recognition of pathogen-associated molecular patterns (PAMPs) by pattern recognition receptors (PRRs). Lp stimulates multiple PRR pathways. During infection in mice, TLR2, TLR4, TLR5, and TLR9 are involved in the recognition of the PAMPs of Lp [9]. Surprisingly, lipopolysaccharide (LPS) from Lp strains is recognized by TLR2, but not the usual TLR4 [10]. On the other hand, a recent study using human macrophages demonstrated that TLR3 and TLR4 were also very important for cytokine production during the process of Lp infection by engaging nucleic acid and LPS, respectively [11]. TLR5 recognizes flagellin and TLR9 recognizes the CpG DNA motif [12]. Lp infection stimulates NOD1 and NOD2, resulting in NF-κB recruitment and subsequent pro-inflammatory cytokine secretion [3]. NAIP5 generates the NLRC4 inflammasome with NLRC4 and is involved in the recognition of bacterial flagellin and components of bacterial type III secretion systems [13]. Lp may translocate RNA into the host cytosol, or an unknown Dot/Icm effector may stimulate the production of RNA intermediates in the host cell, which then stimulates RIG-I/MDA5 signaling and culminates in the production of type Ι interferons (IFNs). However, this signaling does not appear to mediate Lp clearance in vivo [14]. Although it is reported that STING is induced in the lung in response to IFNs in vivo [15], the contribution of the cGAS-STING to in vivo immune responses against Lp has not been studied in detail. To the acquired immunity, both antigen-specific humoral and cell-mediated immune responses are induced during Lp infection. T and B cells are proved to be ultimately required for the clearance of the infection with the immune challenge model [16]. On the other hand, the activation of innate immune cells in the early stages of infection, especially the secretion of cytokines by the activated cells, is a double-edged sword. While limiting the expansion of bacteria, it will also cause different degrees of pathological damage. As in our former report, infection by virulent-strain Lp induced the escalation of systemic pro-inflammation cytokines and caused the death of mice [17].

In addition to respiratory infection, *Legionella* has also been reported to be present in peripheral blood and several other organs [18,19], suggesting that *Legionella* may cause systemic infections. In addition, about 30–50% of LD patients had symptoms of diarrhea. A previous study reported that there were more than 30% of patients (16 out of 42) who had diarrhea as the initial symptom [20]. There was even a report in which a case only had the symptom of diarrhea and no respiratory symptoms throughout the course of the disease [21]. However, direct intestinal infections caused by *Legionella* have been reported very rarely [22], and the mechanism of the enteritis phenotype after *Legionella* pulmonary infection has not been elucidated. Here, we showed that pro-inflammatory cytokines, especially IL-6, play a very important role in the enteritis phenotype induced by Lp pulmonary infection.

## 2. Materials and Methods

### 2.1. Ethics Statement

The mouse experiments in this study were approved by the Research Ethics Committee (APIMCAS2022131) of Institute of Microbiology of Chinese Academy of Sciences (IMCAS). All mouse experimental procedures were performed in accordance with the Beijing Laboratory Animal Welfare and Ethical Guidelines approved by Beijing Administration Committee of Laboratory Animals.

### 2.2. Bacteria, Mice, and Infection

Lp strain sjz088 was isolated from cooling tower water in 2012, as described in our former study [23]. A/J mice were purchased from Nanjing Biomedical Research Institute of Nanjing University (Nanjing, China) and raised at IMCAS animal facility under specific pathogen-free (SPF) conditions. For infection experiments, female mice (6–8 w, 16–19 g) were transferred to a Biosafety Level 2 laboratory. Mice were anaesthetized and inoculated intranasally (i.n.) with about 1 × 10^8^ CFU bacteria in 40 μL sterile PBS [23]. Control mice were given an equal volume of PBS for mock infection. After infections, the bodyweight losses and survival rates of the infected mice were observed daily. In the α-methyltyrosine (MTR) in vivo treatment experiments, MTR was intraperitoneally (i.p.) given to mice at a dose of 60 mg/kg. For each infection experiment, at least 3 mock-infected control and Lp-infected mice were set.

### 2.3. Cell Preparation

For cell preparation, mice from each group were euthanized using the cervical dislocation method under deep anesthesia on 1 day post-infection (dpi), and their intestines were collected for further examination. Lymphocytes from different organs were collected and processed individually. For cells from mesenteric lymph nodes (MLNs) and Peyer’s Patches (PPs), single cell suspensions were obtained using gentle mechanical dissociation in PBS containing 2% FBS. Cells were then washed and resuspended in RPMI 1640 medium supplemented with 10% FBS, 10 mM HEPES, 1 × MEM NEAA (non-essential amino acids), 2 mM L-glutamine, 50 mg/mL streptomycin, 50 U/mL penicillin (all from Gibco, Grand Island, NY, USA), and 50 mM 2-mercaptoethanol (Amresco, Solon, OH, USA) (complete RPMI medium, cRPMI). For intestinal lamina propria lymphocytes (LPLs), after the PPs were removed, the intestine was cut into 2 mm pieces and the epithelium was eliminated using stirring. Intestine pieces were first shaken twice in PBS containing 3 mM EDTA (Sigma-Aldrich, St. Louis, MO, USA) at 37 °C, 160 rpm for 15 min, and then twice in RPMI containing 1% FBS, 1 mM EGTA (Sigma-Aldrich, St. Louis, MO, USA), and 1.5 mM MgCl_2_ for 20 min. Intestine pieces were collected and shaken at 37 °C, 160 rpm for 90 min in RPMI containing 20% FBS, 100 U/mL collagenase, and 5 U/mL DNase 1 (both from Sigma-Aldrich, St. Louis, MO, USA). After the first 60 min and at the end of the incubation, the suspension was dissociated using multiple aspirations through a 10 mL syringe 50 times. The cells were washed and LPLs were purified on a 45%/66.6% discontinuous Percoll (GE HealthCare Technologies Ins., Chicago, IL, USA) gradient at 600× *g* for 20 min. For intestinal intraepithelial lymphocytes (IELs), after the intestine was cut into 2 mm pieces, they were shaken in 199 medium (Gibco, Grand Island, NY, USA) at 37 °C, 160 rpm for 30 min, followed by another 30 min in cRPMI medium. IELs were also finally purified with the 45%/66.6% discontinuous Percoll gradient at 600× *g* for 20 min. As for the intestinal epithelial cells (IECs), intestine was cut along the longitudinal axis; then, the remaining contents were gently washed away in DPBS. After incubation in DPBS supplied with 30 mM EDTA and 1.5 mM DTT on ice for 20 min, intestine samples were transferred into 30 mM EDTA/DPBS and shaken at 37 °C at 160 rpm for 10 min. After intensive vibration, intestine tissue was removed and the cells were washed and resuspended into cRPMI for the further use.

### 2.4. Antibodies and Reagents

For flow cytometric analysis of lymphocytes, the following anti-mouse antibodies were used: anti-CD3 (eBio500A2, PE), anti-DX5 (HMa2, FITC), anti-Ly-6G (RB6-8C5, FITC), and anti-F4/80 (BM8, APC) (all from Invitrogen, Carlsbad, CA, USA); anti-TCR γ/δ (GL3, APC) and anti-CD11b (M1/70, APC/Cyanine7) (both from BioLegend, San Diego, CA, USA); and anti-CD11c (N418, PE, Sungene Biotech Co., Ltd., Tianjin, China). For Western blot analysis, primary antibodies anti-mouse caspase 1 (Casper-1, AdipoGen, San Diego, CA, USA), anti-caspase 3 (Cell Signaling Technology, Danvers, MA, USA), anti-mouse caspase 11 (17D9, Invitrogen, Carlsbad, CA, USA), anti-p53 (ABclonal, Wuhan, China), anti-Bcl-2 (Bioworld, Nanjing, China), and anti-mouse β-actin (6G3, Sungene Biotech Co., Ltd., Tianjin, China) were used; and secondary antibodies goat anti-mouse/rabbit/rat horseradish peroxidase-conjugated antibodies (Jackson ImmunoResearch Laboratories, West Grove, PA, USA) were used. Tocilizumab (anti-IL-6R, Selleck, Houston, TX, USA) was i.p. injected with a dose of 5 mg/kg at 12 h before infection to block IL-6 function. To neutralize IFN-γ, an anti-mouse IFN-γ antibody (R4-6A2; Invitrogen, Carlsbad, CA, USA) was i.p. inoculated with a dose of 250 mg per mouse at 12 h before infection. Naive sera for each antibody were used as a control in each experiment. For the IECs’ ex vivo stimulation, recombinant mouse IL-1α, IL-6, TNF-α, and IFN-γ (all from PeproTech, Rocky Hill, NJ, USA) were used.

### 2.5. FACS Analysis

For flow cytometric analysis of each type of cell in intestine, up to 2 × 10^6^ prepared cells were stained with surface molecules at 4 °C for 30 min. All antibodies were used as 0.1 μg per sample in a 50 μL working system. Stained cells were analyzed with a LSRFortessa flow cytometer (BD Biosciences, San Jose, CA, USA). At least 1.5 × 10^4^ live cells were collected for analysis in each sample. Each type of cell was gated as follows: NK cells, CD3^−^DX5^+^; NKT cells, CD3^+^DX5^+^; γδT cells, CD3^+^TCRγδT^+^ in lymphocytes; and DCs, CD11c^+^Ly-6G^−^F4/80^−^; macrophages, CD11b^+^F4/80^+^; neutrophils, Ly-6G^+^F4/80^−^ in myeloid cells. Data were analyzed with FlowJo software (version 7.6.2, BD Biosciences, San Jose, CA, USA).

### 2.6. Histopathology

For histological analysis, intestine was collected and fixed with 4% paraformaldehyde, dehydrated in a series of graded alcohols, and embedded in paraffin. Tissue sections (5 μm) were cut and stained with hematoxylin and eosin (H&E).

### 2.7. Western Blot

After separation, IECs lysate was prepared using cell lysis buffer (Cell Signaling Technology, Danvers, MA, USA) with 1 mM of phenylmethanesulfonyl fluoride (PMSF, Beyotime, Shanghai, China) and protease inhibitor cocktail (CWBio, Taizhou, China) added on ice. Total protein concentration was measured with a BCA protein assay kit (Gene-Protein Link, Beijing, China). Equal amounts of protein were then loaded onto SDS-polyacrylamide gels for separation. The proteins were subsequently transferred to and immobilized on Immobilon^®^-P PVDF membranes (Merck Millipore, Billerica, MA, USA). Primary antibodies, anti-mouse caspase 1 (1:1000), anti-caspase 3 (1:1000), anti-mouse caspase 11 (1:1000), anti-p53 (1:1000), anti-Bcl-2 (1:2000), and anti-mouse β-actin (1:2000), along with secondary antibodies (1:2000), were used. All antibodies were diluted in 5% (*w*/*v*) non-fat milk in TBS with 0.05% Tween-20 (TBS-T). Protein detection was carried out using Pierce™ ECL Western Blotting Substrate (Thermo Fisher Scientific, Waltham, MA, USA) and visualized with an automatic luminescence imaging system (Tanon, Shanghai, China). For the statistical analysis of Western blot, caspase 1, 3, and 11 were reflected by the ratio of activated fragment to full-length (caspase 1, and 3)/pro-caspase form (caspase 11), and each band of Bcl-2 and p53 was normalized to actin of the same sample, respectively.

### 2.8. Ex Vivo IECs Stimulation

Separated IECs were incubated with recombinant mouse IL-1α (1000 ng/mL), IL-6 (1000 ng/mL), TNF-α (1000 ng/mL), and IFN-γ (10^3^ U/mL) at 37 °C, with 5% CO_2_ for 24 h. After stimulation, IECs were collected, cell lysate was made, and Western blot was performed to check the caspase 3 activation status.

### 2.9. Statistical Analysis

Significant differences between experimental groups were determined using Student’s *t*-test and analysis of variance (ANOVA) with the GraphPad Prism software package (version 8.0.1, GraphPad Software Inc.).

## 3. Results

### 3.1. Construction of a Mouse Model with Syndrome of Enteritis Caused by Lp Pulmonary Infection

**Figure 1:**
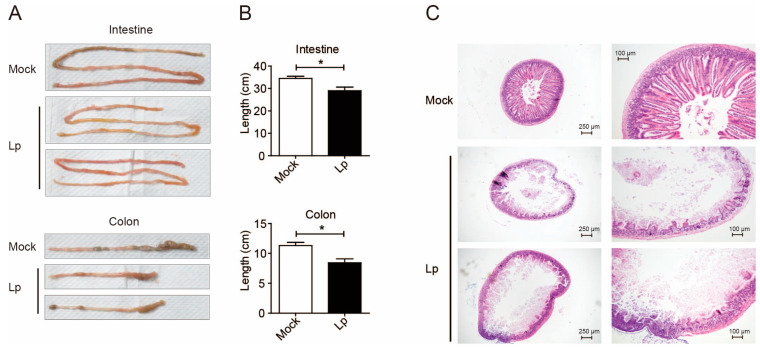
Lp pulmonary infection caused the enteritis phenotype. Pictures (**A**) and statistical results of length (**B**) of intestine (upper) and colon (lower) from mock- and Lp-infected (1 dpi) mice. (**C**) H&E staining of intestine from mock- or Lp-infected mice. *, *p* < 0.05.

In our previous study, a total of 11 6–8-week-old mice were i.n. infected with the same dose of sjz088 in two independent experiments (one for 8 mice, the other for 3 mice). At that time, all Lp-infected mice presented significant clinical signs, including torpor, bodyweight losses, diarrhea, and all the infected mice died within 5 dpi [23]. Therefore, in the present study, the infected mice were euthanized at 1 dpi for pathological examination and cell separation, by which time they had presented significant clinical syndromes. The enteritis phenotype was clearly found during the gross pathological examination. The length of both the colon and small intestines was significantly shortened (Figure 1A,B). In addition, the H&E staining of the small intestine showed obvious pathological changes, which were manifested by the disappearance of intestinal villi and the destruction of the basic structure of the intestine (Figure 1C).

### 3.2. There Was No Significant Change in the Composition of Innate Immune Cells in the Enteritic Phenotype

**Figure 2:**
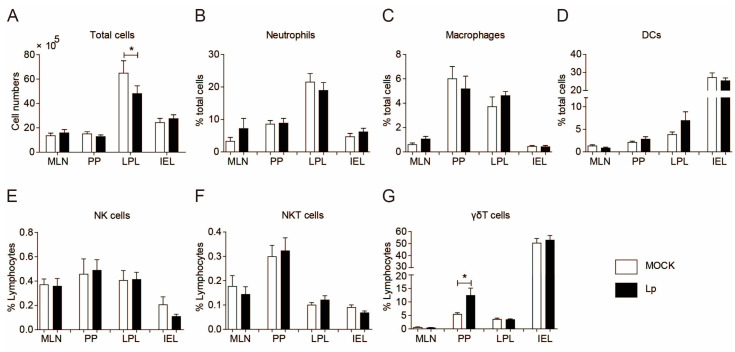
Lp infection did not cause significant changes in the composition of innate immune cells in intestinal tissue. Cell composition of MLN, PP, LPL, and IEL from mock- or Lp-infected (1 dpi) mice was analyzed using flow cytometry. Total cell numbers (**A**); the percentage of neutrophils (**B**), macrophages (**C**), and DCs (**D**) of total cells; and the percentage of NK (**E**), NKT (**F**), and γδT cells (**G**) of lymphocytes are shown. *, *p* < 0.05.

In order to find out the cause of enteritis, we first analyzed the composition of immune cells in the small intestine, including the immune cells from the MLNs, PPs, LPLs, and IELs. Because the strong-virulence strain we used could kill mice in a very short period of time (usually within 5 dpi), and the phenotype of enteritis could be observed on 1 dpi, we focused our attention on innate immune cells. On 1 dpi, except for a slight decrease in the number of cells in the LPLs, there was no significant changes in the number of cells in each fraction (Figure 2A). In each group of innate immune cells, there was no changes in the composition of neutrophils, macrophages, DCs, NK cells, and NKT cells from each fraction (Figure 2B–F). The percentage of γδT cells in PPs doubled after infection (Figure 2G). However, given the low number of these γδT cells in the PPs (γδT cell numbers in PPs were only about 18.3% of that of LPLs, and 2.1% of that of IELs in naïve mice), this increase might not be a significant cause of the enteritis phenotype.

### 3.3. Lp Infection Activated the Caspase 3 in Intestine Epithelial Cells

**Figure 3:**
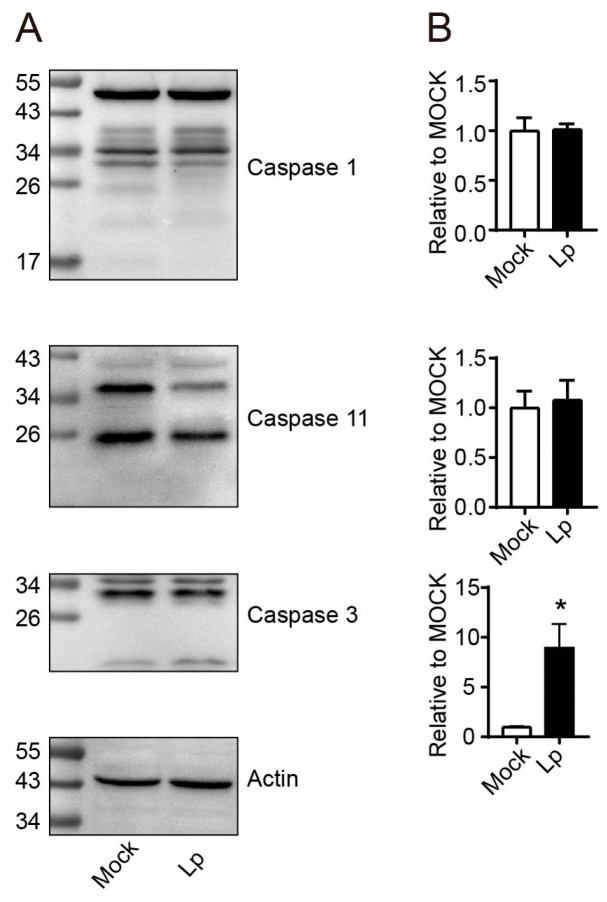
Lp pulmonary infection activated caspase 3 in IECs. The activation status of caspase 1, 3, and 11 was analyzed using Western blot (**A**). The activation of caspase 1 was reflected by the ratio of activated fragment to full-length of mouse caspase 1 ((**B**), upper); the activation of caspase 11 was reflected by the ratio of activated fragment to pro-caspase form ((**B**), middle); the activation of caspase 3 was reflected by the ratio of activated fragment to full-length of mouse caspase 3 ((**B**), lower). All data in B correspond to the mean ± standard error of three independent experiments. *, *p* < 0.05.

Considering that the composition of immune cells failed to explain the cause of enteritis phenotype, we turned to the explanation of the mechanisms under enteritis formation by exploring the cause of the death of IECs. We examined three types of caspases, caspase 1, 3, and 11, which played key roles in causing cell death in mice. As shown in Figure 3, after Lp infection, only caspase 3 was activated in IECs. Therefore, the phenotype of enteritis after Lp infection was formed by cell death caused by the activation of caspase 3 in IECs.

### 3.4. Inhibition of Systemic Inflammation Effectively Alleviated the Enteritis Phenotype Through Downregulating the Activation of Caspase 3

**Figure 4:**
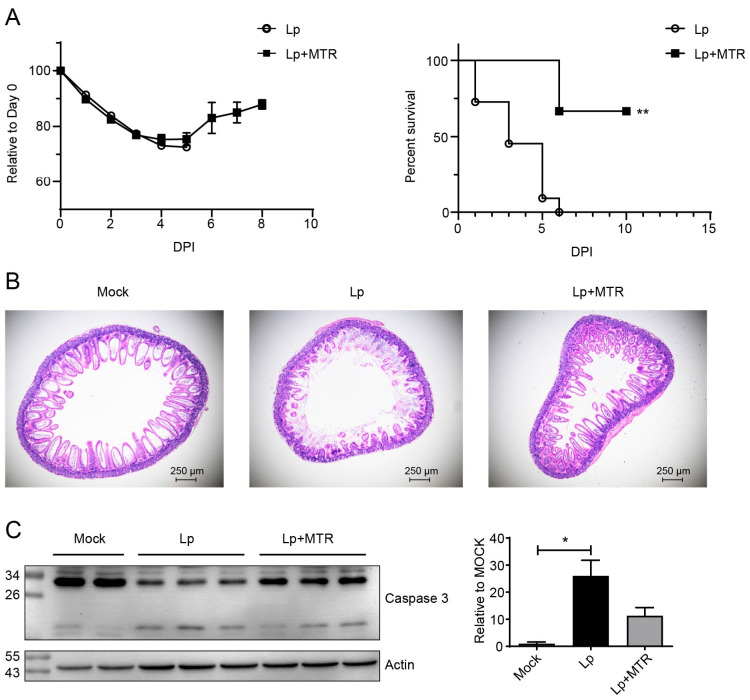
Inhibition of systemic inflammatory cytokine expression alleviated the enteritis phenotype. Bodyweight changes ((**A**), left), survival rates ((**A**), right), and H&E staining of intestines (**B**) of Lp-infected (1 dpi) mice with/without MTR treatment are shown. (**C**) Western blot results show caspase 3 of IECs from Lp-infected (1 dpi) mice with/without MTR treatment (left), and the statistical analysis results of the activation of caspase 3 in the Western blot bands (right) were analyzed using the ratio of activated fragment to full-length of mouse caspase 3. *, *p* < 0.05, **, *p* < 0.01.

Next, we tried to study why an Lp pulmonary infection could cause the activation of caspase 3 in IECs. In a previous study, we reported that an infection with the Lp strain with strong virulence triggered systemic inflammation and increased the levels of inflammatory cytokines in the infected mice sera [17]. Therefore, we then investigated whether there was a correlation between the systemic inflammatory response and the enteritis phenotype. Since a study had reported that MTR could reduce the systemic cytokine release through the direct inhibition of catecholamine synthesis [24], we used MTR to inhibit the inflammatory cytokine production after the Lp infection. The MTR treatment did not change the bodyweight at the early stage after infection; however, it increased the survival rate of the infected mice (Figure 4A). The results of H&E staining showed a significant reduction in IECs losses and maintenance of the intestine basic structures (Figure 4B and Figure S1A). Western blot results showed that MTR treatment could significantly reduce the activation of caspase 3 (Figure 4C). Taken together, the systemic inflammation caused by Lp infection could activate the caspase 3 in IECs, thus playing an important role in the enteritis phenotype after Lp infection.

### 3.5. Lp Infection Induced Expression of IL-6 and IFN-γ That Caused Apoptosis of IECs by Activating Caspase 3 Signal

**Figure 5:**
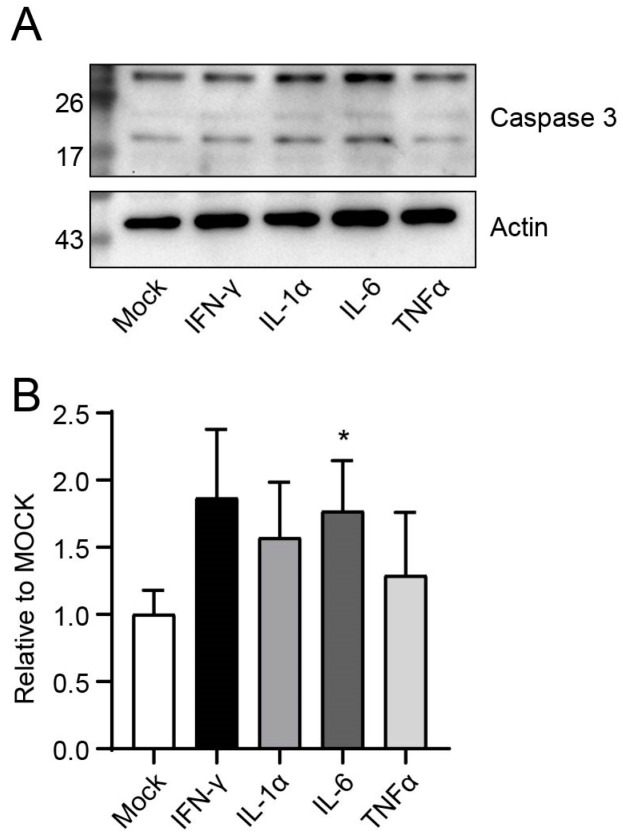
High dose of IL-6 and IFN-γ activated caspase 3 of IECs ex vivo. After purification, IECs were co-cultured with recombinant mouse IL-1α, IL-6, TNF-α, and IFN-γ at 37 °C, 5% CO_2_ for 24 h. (**A**) Western blot results; (**B**) statistical results. Data in B correspond to the mean ± standard error of three independent experiments. *, *p* < 0.05, compared with mock-infected control.

To determine which cytokine played an important role in the enteritis phenotype, we performed an ex vivo stimulation experiment of IECs. After isolation, we stimulated IECs ex vivo with a high dose of various cytokines, which were demonstrated in our previous study to be upregulated in mouse serum after Lp infection [17]. As shown in Figure 5, IECs showed a more significant activation of caspase 3 after a high dose of IFN-γ or IL-6 stimulation compared to other groups. In particular, IL-6 reached statistical significance.

**Figure 6:**
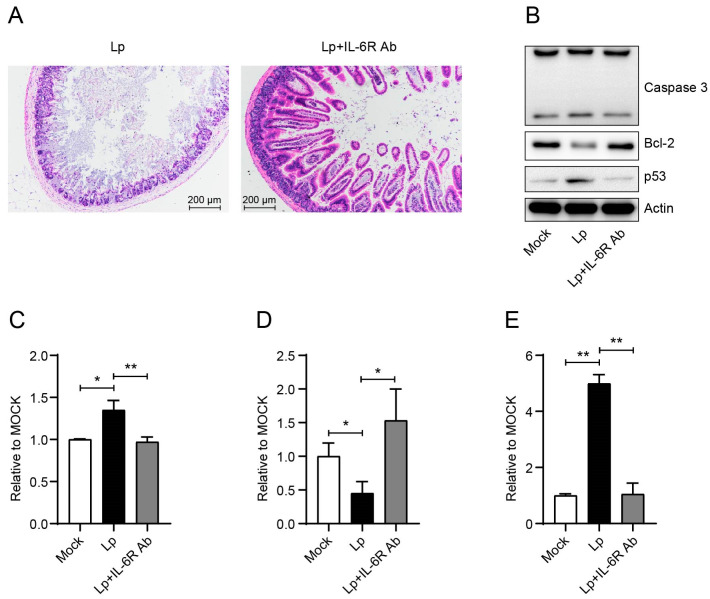
IL-6 played an important role in the enteritis phenotype through p53-Bcl-2-caspase 3 axis. (**A**) H&E staining results show the intestines after Lp infection (1 dpi) with/without IL-6R Ab administration. (**B**) Western blot results show caspase 3, Bcl-2, and p53 of IECs from mock- or Lp-infected mice (1 dpi) with/without IL-6R Ab administration. (**C**–**E**) show the statistical results of caspase 3 (**C**), Bcl-2 (**D**), and p53 (**E**), respectively. Data in (**C**–**E**) correspond to the mean ± standard error of three independent experiments. *, *p* < 0.05, **, *p*< 0.01.

Next, we investigated the role of IL-6 in the enteritis phenotype. After treatment with IL-6R Ab to block the function of IL-6, the symptoms of enteritis obviously reduced base on the H&E staining of intestine tissue (Figure 6A and Figure S1B). Meanwhile, IL-6R Ab reduced the activation of caspase 3 to the same level of the mock group (Figure 6B,C). Also, from Figure 6 we can see that Lp infection decreased the level of anti-apoptotic family member Bcl-2 (Figure 6B,D), while it upregulated p53 expression (Figure 6B,E) in IECs. IL-6R Ab could reverse these changes, and pull these factors back to the mock level (Figure 6B–E).

**Figure 7:**
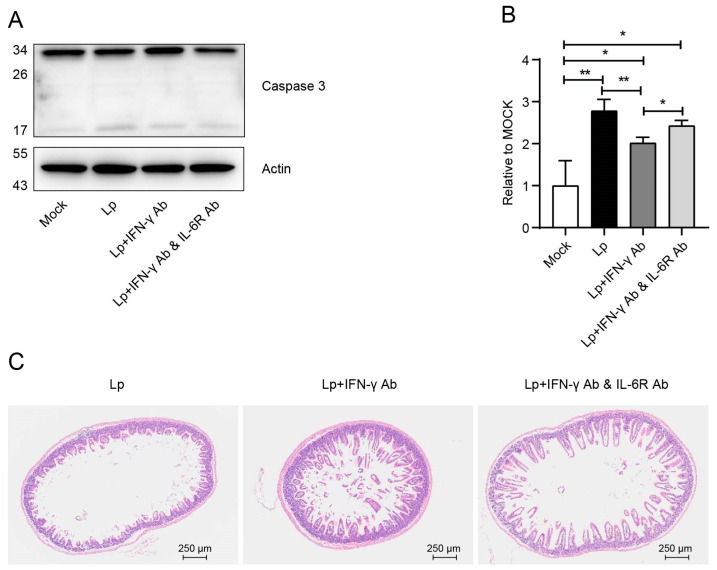
IFN-γ played an important role in the enteritis phenotype. (**A**) Western blot results show caspase 3 of IECs from mock- or Lp-infected mice (1 dpi) with/without treatment of anti-IFN-γ Ab or anti-IFN-γ Ab + IL-6R Ab. (**B**) Statistical results of Western blot. (**C**) H&E staining results of Lp-infected mice (1 dpi) with/without treatment of anti-IFN-γ Ab or anti-IFN-γ Ab + IL-6R Ab. Data in B correspond to the mean ± standard error of three independent experiments. *, *p* < 0.05, **, *p* < 0.01.

On the other hand, IFN-γ was reported to induce non-pyroptotic and non-apoptotic cell death in *Salmonella*-infected IECs [25]; we also demonstrated in our former study that it could induce thymocytes apoptosis in the thymus during influenza virus infection [26]. More importantly, our results showed that IFN-γ activated caspase 3 in IECs using our ex vivo stimulation system (Figure 5). Therefore, we detected the influence of IFN-γ on the enteritis phenotype during Lp infection. The neutralization of IFN-γ significantly downregulated the activation of caspase 3 in IECs (Figure 7A,B). Through the results of H&E staining, we found that neutralizing IFN-γ significantly improved the symptoms of enteritis and kept the structure of the small intestine intact (Figure 7C and Figure S1C). As a conclusion, IL-6 and IFN-γ caused apoptosis of IECs by activating caspase 3, resulting in the enteritis phenotype.

## 4. Discussion

Diarrhea caused by Lp pulmonary infection is an important symptom of LD and is expected to be used as an adjunct diagnosis for Lp infection. However, the unclear mechanisms of the pathogenesis affect the clinical diagnosis and treatment, and may even cause misdiagnosis. Here, we constructed a mouse model of enteritis caused by pulmonary infection with a strong-virulence strain of Lp; based on this model we demonstrated that the systemic inflammatory cytokines, such as IL-6, could activate caspase 3, through upregulating the expression of p53 and inhibiting the expression of Bcl-2, leading to the enteritis phenotype. This also explained why, in our research, only the strong-virulence strain infection could cause the enteritis phenotype, but not weak ones. Although after pulmonary infection by either strong- or weak-virulence strains, live bacteria could be isolated from the feces of the infected mice (unpublished data), only a strain with strong virulence could stimulate the release of inflammatory cytokines into the peripheral blood during infection [17].

As an atypical respiratory pathogen, whether Lp could cause enteritis through directly infecting the intestinal tract is uncertain. In previous studies, only one report described the detection of Lp using direct immunofluorescence staining in the colon following colectomy in a patient with ulcerative colitis [22]. However, the same research team did not re-detect intestinal infection with Lp in subsequent and larger screenings of patients with diarrhea [27]. Lp may enter the intestine through the digestive system, circulatory system, or other routes. In our research, we found that direct gavage to the same amount of Lp neither caused infection nor showed any symptoms in the mouse model (unpublished data). This result suggested that lung infection might be necessary to cause the enteritis phenotype before Lp reached the intestine. On the other hand, since Lp was reported to be isolated from blood [18], it might reach the intestinal tract through blood circulation. However, considering the very few cases of direct intestinal infection by Lp, further research is needed on whether Lp could directly infect the intestinal tract and, thus, cause enteritis.

In our study, although we confirmed the importance of inflammatory cytokines in the enteritis phenotype caused by Lp pulmonary infection (Figure 4, Figure 6 and Figure 7), and there was no significant change in the composition of immune cells in the gut-related tissues (Figure 2), we could not rule out that some kinds of immune cells might play some roles in the development of the enteritis phenotype through functional changes. In particular, the proportion of γδT cells in PPs was doubled after infection. While we did not conduct research on this due to the small number of immune cells in PPs compared to other tissues, it warrants to study in the future because of its multiple immunomodulatory functions.

In summary, we clearly revealed for the first time that inflammatory cytokines, especially IL-6, downregulated the expression of Bcl-2 through the p53 signaling pathway during Lp pulmonary infection, thereby promoting the apoptosis of IECs with the activation of caspase 3, and ultimately causing the enteritis phenotype. This study provides a theoretical basis and experimental data support for the more accurate diagnosis and treatment of Lp infection in clinical practice.

## Data Availability

The data that support the findings of this study are available from the corresponding authors upon reasonable request.

## Supplementary Materials

The following supporting information can be downloaded at: https://www.mdpi.com/article/10.3390/microorganisms13020313/s1, Figure S1: The histopatholog-ical changes in intestines of the Lp-infected mice treated with different agents.

## References

[1] Fraser D.W., Tsai T.R., Orenstein W., Parkin W.E., Beecham H.J., Sharrar R.G., Harris J., Mallison G.F., Martin S.M., Mcdade J.E. (1977). Legionnaires disease—Description of an epidemic of pneumonia. N. Engl. J. Med..

[2] Diederen B.M.W. (2008). *Legionella* spp. and Legionnaires’ disease. J. Infect..

[3] Brown A.S., Yang C., Hartland E.L., van Driel I.R. (2017). The regulation of acute immune responses to the bacterial lung pathogen *Legionella pneumophila*. J. Leukoc. Biol..

[4] Mondino S., Schmidt S., Rolando M., Escoll P., Gomez-Valero L., Buchrieser C. (2020). Legionnaires’ disease: State of the art knowledge of pathogenesis mechanisms of *Legionella*. Annu. Rev. Pathol.-Mech..

[5] Correia A.M., Gonçalves J., Gomes J.P. (2016). Probable person-to-person transmission of Legionnaires’ disease. N. Engl. J. Med..

[6] Jamieson A.M., Pasman L., Yu S., Gamradt P., Homer R.J., Decker T., Medzhitov R. (2013). Role of tissue protection in lethal respiratory viral-bacterial coinfection. Science.

[7] Cassell K., Davis J.L., Berkelman R. (2021). Legionnaires’ disease in the time of COVID-19. Pneumonia.

[8] Phin N., Parry-Ford F., Harrison T., Stagg H.R., Zhang N., Kumar K., Lortholary O., Zumla A., Abubakar I. (2014). Epidemiology and clinical management of Legionnaires’ disease. Lancet Infect. Dis..

[9] Park B., Park G., Kim J., Lim S.A., Lee K.M. (2017). Innate immunity against *Legionella pneumophila* during pulmonary infections in mice. Arch. Pharm. Res..

[10] Girard R., Pedron T., Uematsu S., Balloy V., Chignard M., Akira S., Chaby R. (2003). Lipopolysaccharides from *Legionella* and *Rhizobium* stimulate mouse bone marrow granulocytes via Toll-like receptor 2. J. Cell Sci..

[11] Grigoryeva L.S., Cianciotto N.P. (2021). Human macrophages utilize a wide range of pathogen recognition receptors to recognize *Legionella pneumophila*, including Toll-Like Receptor 4 engaging Legionella lipopolysaccharide and the Toll-like Receptor 3 nucleic-acid sensor. PLoS Pathog..

[12] Massis L.M., Zamboni D.S. (2011). Innate immunity to *Legionella pneumophila*. Front. Microbiol..

[13] Lightfield K.L., Persson J., Brubaker S.W., Witte C.E., von Moltke J., Dunipace E.A., Henry T., Sun Y.H., Cado D., Dietrich W.F. (2008). Critical function for Naip5 in inflammasome activation by a conserved carboxy-terminal domain of flagellin. Nat. Immunol..

[14] Monroe K.M., McWhirter S.M., Vance R.E. (2009). Identification of host cytosolic sensors and bacterial factors regulating the type I interferon response to *Legionella pneumophila*. PLoS Pathog..

[15] Naujoks J., Tabeling C., Dill B.D., Hoffmann C., Brown A.S., Kunze M., Kempa S., Peter A., Mollenkopf H.J., Dorhoi A. (2016). IFNs modify the proteome of *Legionella*-containing vacuoles and restrict infection via IRG1-derived itaconic acid. PLoS Pathog..

[16] Kikuchi T., Andarini S., Xin H., Gomi K., Tokue Y., Saijo Y., Honjo T., Watanabe A., Nukiwa T. (2005). Involvement of fractalkine/CX3CL1 expression by dendritic cells in the enhancement of host immunity against *Legionella pneumophila*. Infect. Immun..

[17] Wang H., Lu J., Li K., Ren H., Shi Y., Qin T., Duan X., Fang M. (2018). The virulence of *Legionella pneumophila* is positively correlated with its ability to stimulate NF-kappaB activation. Future Microbiol..

[18] Edelstein P.H., Meyer R.D., Finegold S.M. (1979). Isolation of *Legionella pneumophila* from blood. Lancet.

[19] Evans C.P., Winn W.C. (1981). Extrathoracic localization of *Legionella pneumophila* in Legionnaires pneumonia. Am. J. Clin. Pathol..

[20] Lattimer G.L., Ormsbee R.A. (1981). Clinical Features in Legionnaires’ Disease.

[21] Dalal N., Athwal P.S.S., Tharu B., Shah P., Shah L. (2020). Legionnaires disease presenting as diarrhea: A case report. Cureus.

[22] Schmidt T., Pfeiffer A., Ehret W., Keiditsch E., Ruckdeschel G., Kaess H. (1989). *Legionella* infection of the colon presenting as acute attack of ulcerative-colitis. Gastroenterology.

[23] Qin T., Zhao D., Zhu L., Ren H., Li Y., Liu X., Li X., Li W., Zhao N., Lu J. (2022). *Legionella pneumophila* risk from cooling tower systems in China. Appl. Environ. Microbiol..

[24] Staedtke V., Bai R.Y., Kim K., Darvas M., Davila M.L., Riggins G.J., Rothman P.B., Papadopoulos N., Kinzler K.W., Vogelstein B. (2018). Disruption of a self-amplifying catecholamine loop reduces cytokine release syndrome. Nature.

[25] Ingram J.P., Tursi S., Zhang T., Guo W., Yin C.R., Wynosky-Dolfi M.A., van der Heijden J., Cai K.Q., Yamamoto M., Finlay B.B. (2018). A nonpyroptotic IFN-γ-triggered cell death mechanism in nonphagocytic cells promotes in vivo. J. Immunol..

[26] Duan X.F., Lu J., Zhou K., Wang J., Wu J.H., Gao G.F., Fang M. (2015). NK-cells are involved in thymic atrophy induced by influenza A virus infection. J. Gen. Virol..

[27] Schmidt T., Pfeiffer A., Ehret W., Keiditsch E., Ruckdeschel G., Kaess H. (1990). *Legionella* infection in ulcerative-colitis. Gastroenterology.

