# Supplementary material for: IL-6–Caspase 3 Axis Plays an Important Role in Enteritis Caused by Legionella pneumophila Pulmonary Infection
Source: Microorganisms. 2025 Feb 1;13(2):313. doi: 10.3390/microorganisms13020313 (PMC11858493; doi:10.3390/microorganisms13020313)
Supplement: Supplementary file 1 [file microorganisms-13-00313-s001.zip › Figure S1-L.pdf]

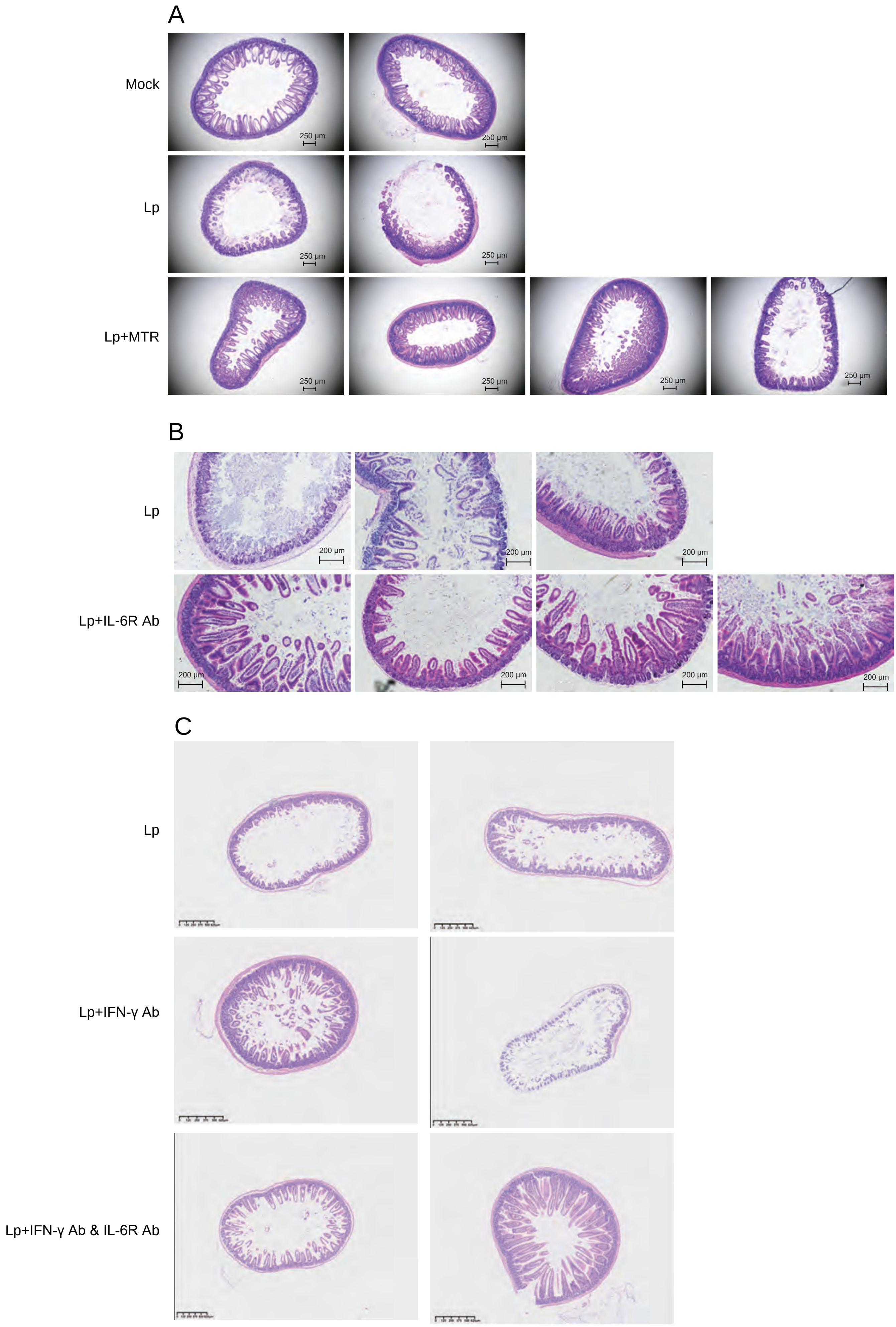

Figure S1: The histopathological changes in intestines of the Lp infected mice treated with different agents.
A, showed the H&E pictures of mice with mock infection (upper), Lp infection (middle), or Lp infection with the MTR treatment (lower).
B, showed the H&E pictures of Lp infected mice with (lower)/without (upper) IL-6R Ab treatment.
C, showed the H&E pictures of Lp infected mice treated with control sera (upper), anti-IFN-γ Ab (middle), or anti-IFN-γ Ab + IL-6R Ab (lower).
